# Empirical food webs of 12 tropical reservoirs in Singapore

**DOI:** 10.3897/BDJ.10.e86192

**Published:** 2022-09-14

**Authors:** Clare Wilkinson, Rayson B H Lim, Jia Huan Liew, Jeffrey T B Kwik, Claudia L Y Tan, Tan Heok Hui, Darren C J Yeo

**Affiliations:** 1 National University of Singapore, Singapore, Singapore National University of Singapore Singapore Singapore; 2 Lingnan University, Hong Kong, China Lingnan University Hong Kong China; 3 Lee Kong Chian Natural History Museum, National University of Singapore, Kent Ridge, Singapore Lee Kong Chian Natural History Museum, National University of Singapore Kent Ridge Singapore

**Keywords:** gut content, stable isotope analysis, freshwater communities, reservoirs, trophic interactions

## Abstract

**Background:**

Food webs summarise trophic interactions of the biotic components within an ecosystem, which can influence nutrient dynamics and energy flows, ultimately affecting ecosystem functions and services. Food webs represent the hypothesised trophic links between predators and prey and can be presented as empirical food webs, in which the relative strength/importance of the respective links are quantified. Some common methods used in food web research include gut content analysis (GCA) and stable isotope analysis (SIA). We combine both methods to construct empirical food web models as a basis for monitoring and studying ecosystem-level outcomes of natural (e.g. species turnover in fish assemblage) and intentional environmental change (e.g. biomanipulation).

**New information:**

We present 12 food webs from tropical reservoir communities in Singapore and summarise the topology of each with widely-used network indices (e.g. connectance, link density). Each reservoir was surveyed over 4–6 sampling occasions, during which, representative animal groups (i.e. fish species and taxonomic/functional groups of zooplankton and benthic macroinvertebrates) and all likely sources of primary production (i.e. macrophytes, periphyton, phytoplankton and riparian terrestrial plants) were collected. We analysed gut content in fishes and bulk isotope (d^13^C and d^15^N) profiles of all animals (i.e. fishes and invertebrates) and plants collected. Both sets of information were used to estimate the relative strength of trophic relationships using Bayesian mixing models. We document our protocol here, alongside a script in the R programming language for executing data management/analyses/visualisation procedures used in our study. These data can be used to glean insights into trends in inter- and intra-specific or guild interactions in analogous freshwater lake habitats.

## Introduction

Food webs depict feeding interactions in an ecosystem; they are indicative of energy flow between species and/or communities through ecosystems (Garvey and Whiles 2016, Barnes et al. 2018) and are shaped by evolutionary, ecological and neutral processes (Akin and Winemiller 2006). Food webs can, therefore, be used to study a range of topics, ranging from: predator-prey and competitive relationships, to the web network including food chain length, connective and potential regulation via top-down or bottom-up control (Winemiller and Polis 1996).

Gut content analysis (GCA), theoretical models based on data from literature and allometric scaling were common methods used in earlier food web studies (e.g. Cohen 1978, Pimm 1982), but technological advances have enabled the construction of more precise, empirical food webs. More contemporary or newer methods include bulk stable isotope analysis (SIA) and compound specific isotope analysis (CSIA) of specific molecules (e.g. amino acids or fatty acids; Arrington et al. 2006, Grey 2006, Chikaraishi et al. 2014, Liew et al. 2019), of which, bulk SIA is one of the most widely used, given its accessibility and relative affordability. These approaches provide a quantitative measure of resource assimilation (as opposed to ingestion measured by gut contents analysis) and allow ecologists to assess the relative strength of trophic pathways in a food web (Michener and Lajtha 2007). Food web construction informed by SIA is based on the differential ratios of naturally-occurring carbon-13 and nitrogen-15 isotopes, expressed as δ^13^C (‰) and δ^15^N (‰), respectively. The former is indicative of contributions from different carbon (i.e. food) sources, while the latter correlates with trophic position (Hopkins and Ferguson 2012, Parnell 2020), thereby yielding insights into resource-consumer links and roles within the community.

As part of an in-depth study of 12 reservoirs in the tropical island nation of Singapore, we combined two complementary methods: GCA and SIA, to elucidate the empirical food web structure of the freshwater reservoir communities. These systems constitute novel ecosystems (Hobbs et al. 2009) which are typically dominated by a mix of disturbance-tolerant native and non-native species (Yeo and Lim 2011, Liew et al. 2016, Tan et al. 2020) and this represents the first study to characterise and quantify the trophic relationships of the reservoir communities in Singapore.

## General description

### Purpose

The aim of this paper is to provide the most comprehensive dataset to date, describing trophic interactions between key plant and animal groups in 12 of Singapore’s 17 reservoirs. The data were constructed using a standardised procedure utilising gut content and stable isotope data, which we detail in the following sections. This facilitates unbiased comparisons of food web indices (e.g. fraction of basal producers, intermediate, top predators; mean Pianka’s overlap; niche breadth), allowing researchers to address questions pertaining to natural (e.g. environmental) and artificial (e.g. urbanisation) drivers of food web trends across spatial or temporal gradients.

## Project description

### Title

Project 1 (Dec 2014–May 2016): Biodiversity and biological interactions in six Singapore reservoirs — a pilot study of food web and trophic structure and implications for environmental and water quality management.

Project 2 (June 2016–Oct 2018): Biodiversity and biological interactions in Singapore’s reservoirs and waterways — a study of food web and trophic structure and implications for environmental and water quality management.

### Personnel

Darren C J Yeo, Heok Hui Tan, Timothy Jardine, Jeffrey T B Kwik, Rudolf Meier, Jia Huan Liew, Clare Wilkinson, Rayson B H Lim, Claudia L Y Tan, Ming Li Chen, Wen Qing Ng, Yvonne Y W Kwang, Abel C Y Saw and Shan Shan Liu

### Study area description

**Figure 1.**
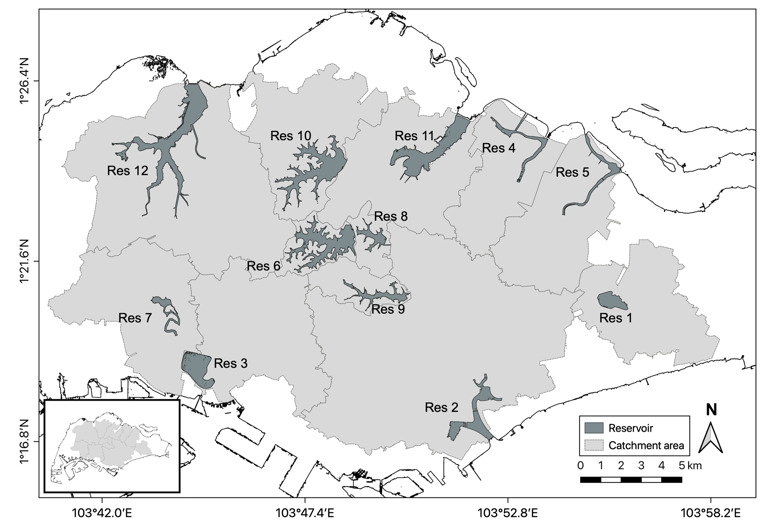
Location of the 12 reservoirs sampled in Singapore.

The data presented in this paper were collected from 12 man-made reservoirs in the tropical island of Singapore (1°21.0' N, 103°49.11' E; Fig. 1, Table 1), six being sampled in Project 1 and six sampled in Project 2. The reservoirs are located within catchments with land-use regimes ranging from protected nature reserves (i.e. riparian vegetation consisting of secondary and primary forests) to urban centres (i.e. riparian zone consisting of rocky rip-rap and impervious surfaces with minimal herbaceous plant cover). The reservoirs vary in age (i.e. time since impoundment), ranging from eight to more than 100 years (Yeo and Lim 2011). The reservoirs are typically characterised by warm (mean water surface temperature of 28.9–31.0°C), slightly turbid and basic (mean pH from 7.1–8.4) waters that are dominated by non-native fish assemblages (Kwik et al. 2020, Tan et al. 2020).

### Funding

This project was funded by PUB, Singapore’s Water Agency [National University of Singapore grant number R-154-000-619-490 and R-154-000-A20-490].

## Sampling methods

### Study extent

Each of the 12 reservoirs were surveyed independently for 2–3 months between December 2014 and October 2018 in Singapore. Our surveys targeted macrofauna (i.e. fish, benthic invertebrates), microfauna (i.e. zooplankton, phytoplankton and periphyton) and riparian vegetation (i.e. C3 and C4 plants) that were broadly representative of the biotic communities observed in each reservoir (Table 2). In total, the 12 food webs sampled constituted 52 fish species and one hybrid (*Oreochromis mossambicus* × *Oreochromis niloticus*), 15 isotopically distinct invertebrate orders, classes or families and nine basal resources.

### Sampling description

Fishes were collected using a combination of cast netting (net dimensions: 4 m radius, 2 cm mesh size), trapping (trap dimensions: 50 cm × 60 cm × 40 cm, 2 cm mesh size) and boat electrofishing (pulsed DC electrofishing, model ETS-MBS-1D-COL) in the littoral zone of each reservoir to optimise sampling coverage across various depths and fish sizes. We performed 10 casts, deployed three traps (for 48 hours) and conducted four 5-minute bursts of electrofishing, per transect. Live specimens were identified to species level (Baker and Lim 2008, Ng and Tan 2010), measured for standard length (cm), weighed for total wet weight (g) using PESOLA weighing scales and up to three individuals per species were euthanised for GCA and SIA. Invertebrate samples were collected using multiple methods. Specifically, benthic invertebrates were collected using two submerged colonisers filled with exogenous substrate (i.e. coconut palm fronds) which were deployed at a fixed depth of 1–1.5 m in the littoral zone for a total of 8–12 weeks at each transect (Loke et al. 2010). Additionally, a hand-held dip net (500 µm mesh size) was used to collect invertebrates from the surface and plankton nets (100 µm mesh size) used to collect zooplankton. All fixed (in 70% ethanol) invertebrate specimens were identified to the highest taxonomic resolution possible, based on identification keys to the freshwater macroinvertebrates of the Malaysian region and of Singapore (Yule and Yong 2004, Blakely et al. 2010). Phytoplankton were also collected by towing a 80 µm and 50 µm mesh size plankton net at the surface for ≈ 100 m per transect. Zooplankton and phytoplankton samples were then separated manually using pipettes or by successive filtration using cell strainers (100 µm, 70 µm and 45 µm mesh size) and the purity and identity of the samples evaluated under a dissecting microscope. Other basal resources were collected: periphyton and epiphytic algal samples were sampled from hard surfaces in the littoral zones; and macrophytes and riparian plants and/or grass samples comprised leaf clippings. 

### Quality control

To facilitate representative sampling, each reservoir was systematically subdivided into six or eight transects (dependent on reservoir size; ≈ 200 m per transect) spanning multiple littoral habitat types (i.e. rocky bund, forested, macrophyte-dominated). Sampling effort and protocols for the various taxonomic groups were standardised to facilitate comparability between reservoirs as described above. In addition, fish species identity was validated by taxon experts from the Lee Kong Chian Natural History Museum.

### Step description

We followed general protocols from Liew et al. (2018) to combine GCA and SIA data to construct empirical food webs for all 12 reservoirs. We used GCA to provide information for the selection of potential prey, before estimating the relative strength of trophic relationships using Bayesian Mixing Models. 

**I. Gut content analysis**

The gut content of 47 fish species with at least four individuals having full gut were analysed to complement the stable isotope analysis (Kwik et al. 2020). In total, 1269 suitable guts were included for the dietary analysis. Dietary items for each fish species were identified under a dissecting microscope in a Petri dish and grouped into ten broad categories: 1) substrate (i.e. inorganic sediment), 2) unidentifiable animal matter (i.e. highly-digested matter of non-fish vertebrates), 3) plant matter (e.g. whole or parts of leaves, fruit and flowers), 4) periphyton (i.e. benthic and filamentous algae mats), 5) phytoplankton (i.e. pelagic algae, e.g. *Microcystis*, *Planktolyngbya* and *Staurastrum*), 6) zooplankton (e.g. Rotifera, Copepoda), 7) insect larvae (i.e. whole or parts of aquatic insect larvae, e.g. Chironomidae, Caenidae), 8) gastropods (i.e. aquatic snails and bivalves, e.g. Viviparidae, Ampullariidae), 9) decapod crustaceans (i.e. crabs and shrimp, e.g. Palaemonidae) and 10) fishes (i.e. whole body, scales or bones). The relative contributions of various dietary items for each fish species were determined, based on their frequency of occurrence (FO_%_) and volumetric contribution (VO_%_) to calculate the feeding index (Sá-Oliveira et al. 2014):

\begin{varwidth}{50in}
        \begin{equation*}
            FI_i = \frac{(FO_i \times VO_i)}{\sum (FO_i \times VO_i)}
        \end{equation*}
    \end{varwidth}

where *FI_i_* = feeding index of species *i*, *FO_i_* = frequency of occurrence of diet item *i* and *VO_i_* = volume of diet item *i*.

**II. Stable isotope analysis**

We estimated the relative strength of trophic interactions of consumers and resources in the food webs using bulk ^13^C/^12^C (i.e. δ^13^C) and ^15^N/^14^N (i.e. δ^15^N) isotope profiles. Tissue samples for primary producers (e.g. riparian plants and macrophytes, phytoplankton, periphyton) consisted of leaf clippings from plants, filtered phytoplankton samples and substrate-free periphyton samples. Invertebrate samples comprised whole organisms for smaller specimens (e.g. dipterans) and muscle tissue for the larger taxa (e.g. gastropods). For fish samples, we extracted muscle tissue from the dorsal region of each individual. We collected a minimum of three samples per taxon and excluded rare species (with less than three individuals collected) from subsequent analyses. However, we made an exception for taxa that were abundant in the study site, but were difficult to isolate for SIA (e.g. Copepoda).

All samples were oven-dried for 48–72 h at 68.5–70.0°C, homogenised, ground to a fine powder and weighed (to the nearest 0.0001 g): 1 mg for consumers (e.g. fish, invertebrates) or 4–5 mg for primary producers (e.g. phytoplankton and plants) following protocols described in Jardine et al. (2003). In total, 5339 tissue samples were prepared and analysed. These consisted of 1042 primary producers, 1476 invertebrates and 2821 fish samples, respectively. These were packed in standard tin capsules and sent to the Stable Isotope Facility at the University of California, Davis for determination of stable isotopic profiles (δ^13^C and δ^15^N). 

**III. Construction of empirical food webs**

We summarised the trophic information derived from GCA and SIA into predation matrices (n = 12). The GCA information was used to inform and/or restrict the pool of potential prey included in the models for each fish species, while information from published literature were used to identify potential resources for the invertebrate taxa. We used Bayesian stable isotope mixing models to estimate the proportional source contribution to diets of consumers by fitting probability models to the isotopic data (e.g. isotopic ratios, elemental concentrations, sample variations and trophic fractionation). 

Before running the mixing models, we corrected the δ^13^C isotopic profiles of samples that comprised whole individuals (e.g. small invertebrates) for lipid-enrichment using procedures described in Logan et al. (2008). We also adjusted the stable nitrogen and carbon isotope data to account for trophic discrimination, using taxon-specific values for δ^15^N (following Bunn et al. 2013) and generic values for δ^13^C (0.4 ± 1.3‰; Post 2002). We note that fractionation values reported in Bunn et al. (2013) were derived from lotic ecosystems, while our study systems are lentic. Nevertheless, the range of fractionation values fall within commonly reported fractionation ranges of 2–4‰, so we did not expect our findings to deviate significantly if we used lentic-specific (but not taxon-specific) values instead. Moreover, strong evidence of inter-taxa differences in δ^15^N fractionation (Sweeting et al. 2007, Bunn et al. 2013) suggest the importance of accounting for taxon-specific variations in isotopic fractionation, especially given the broad taxonomic coverage of our work. We ran 30,000 iterations of each mixing model (i.e. each consumer) with a burn-in of 10,000 draws using Markov Chain Monte Carlo (MCMC) processes on the *simmr *statistical package, version 0.3 (Parnell 2020). Model convergence was assessed using Gelman-Rubin diagnostic parameter (**≈ **1).

We assessed the feasibility of all our mixing models prior to extracting finalised source contribution values using two criteria. First, we ensured that consumer isotopic profiles were bounded within mixing polygons (evaluated by an isoplot produced by the model; Phillips et al. 2014). The mixing polygons were also used to assess isotopic overlap between taxa in a food web. If there was significant isotopic overlap (ANOVA/t-test; p-value < 0.05) between phenotypically similar taxa, taxa were pooled to a higher taxonomic level, for example, genus for fish and to the order or class level for invertebrates, to create isotopically distinct taxonomic units. Second, we ensured that sources (i.e. prey) warranted inclusion in mixing models by removing items which fall below a minimum threshold. We used the following equation to calculate consumer specific threshold values: (1/N)*0.25, where N is number of sources for a consumer. If multiple food sources fall below the threshold value, we deemed them as ‘unimportant’ sources and removed them sequentially (starting from the lowest contributor), while ensuring that the removal of each source does not result in consumer profiles falling outside mixing polygons. We also checked for missing consumer sources by assessing respective mixing polygons, finding that a primary source was missing in three reservoirs where phytoplankton were too scarce to meet the required weight for SIA. For these three reservoirs, we created an ‘unknown producer’ source node using the common practice of adapting isotopic signatures of zooplankton, a likely predator of this missing resource (Grey et al. 2000, Post 2002, Matthews and Mazumder 2003). Finally, we summarised source-contribution for each consumer using the median value of posterior distributions into a predation matrix (column = predator; row = prey). 

**Figure 2.**
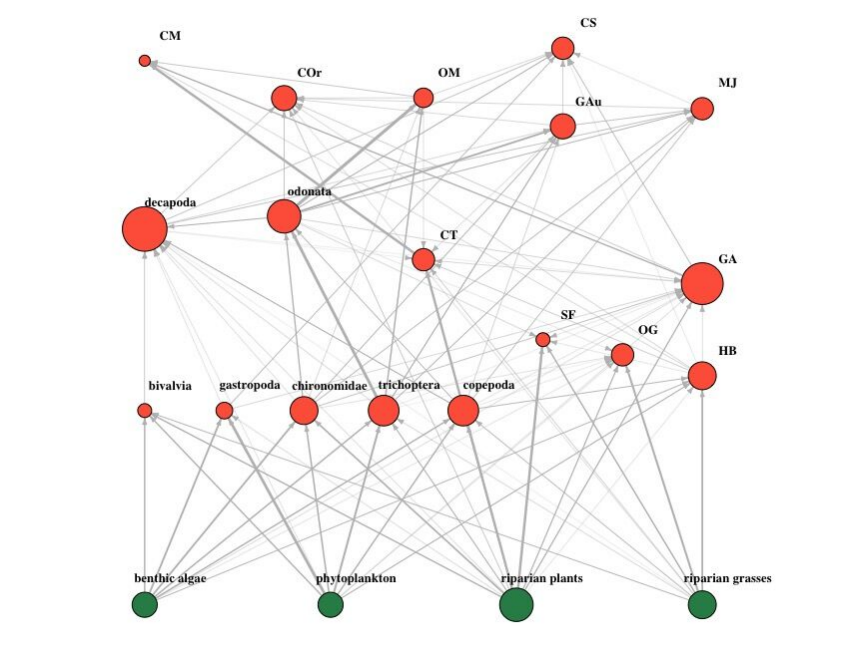
Food web diagram for Reservoir 6. Created from stable isotope data, using the code in Suppl. material 1 and data in Suppl. material 2. The size of each node representing individual taxa is indicative of the number of links formed with other taxa (larger nodes indicate more links), while the thickness of arrows representing predator-prey relationship indicates the relative interaction strengths (thicker arrows indicate greater interaction).

We provide the R script used for producing the empirical food webs, as well as the raw data from one reservoir (res 6) to facilitate replication of our procedure (Suppl. materials 1, 2) and produce a food web (Fig. 2). 

## Geographic coverage

### Description

N.A. (already included in “Study area description”)

## Taxonomic coverage

### Description

Table 2

## Traits coverage

N.A.

## Temporal coverage

### Notes

December 2014 to October 2018

## Usage licence

### Usage licence

Creative Commons Public Domain Waiver (CC-Zero)

## Data resources

### Data package title

Diet composition of the fish species and food web matrix for 12 shallow tropical reservoirs in Singapore.

### Resource link

https://doi.org/10.5061/dryad.jsxksn088

### Number of data sets

4

### Data set 1.

#### Data set name

diet composition_fish.xlsx

#### Data format

Excel spreadsheet

#### Description

This dataset contains information on the diet information fish species with more than four replicates recorded from 12 reservoirs in Singapore.

**Data set 1.** 

Column label	Column description
Reservoir	Identifier for the reservoir.
Latitude	The value, in degrees minutes, of the water body's position north of the equator as determined from Google Earth.
Longitude	The angular distance, in degrees minutes, of the water body's position east of the meridian at Greenwich, England, as determined from Google Earth.
Sampling period	Period during which the fishes were collected from our surveys using electrofishing, traps and cast nets.
Fish species	Scientific name of various fish species collected for gut content analysis.
Replicates	Number of full guts examined.
substrate	The mean proportion of the total gut content volume accounted for by small rocks and sand.
unidentified.animal.matter	The mean proportion of the total gut content volume accounted for by unidentifiable prey items.
plant	The mean proportion of the total gut content volume accounted for by plant materials including leaf fragments, seeds, fruits and woody debris.
periphyton	The mean proportion of the total gut content volume accounted for by benthic and filamentous algae mats.
phytoplankton	The mean proportion of the total gut content volume accounted for by pelagic algae.
zooplankton	The mean proportion of the total gut content volume accounted for by zooplankton including Rotifers as well as from orders Cladocera, Cyclopoida, Harpacticoida and Calanoida.
insect	The mean proportion of the total gut content volume accounted for by terrestrial or aquatic insects.
decapod	The mean proportion of the total gut content volume accounted for by freshwater shrimps, crabs and crayfish.
mollusc	The mean proportion of the total gut content volume accounted for by gastropods and bivalves including shells and operculum.
fish	The mean proportion of the total gut content volume accounted for by fishes, including bones and scales.
detritus	The mean proportion of the total gut content volume accounted for by fine and coarse particulate organic matter.
hirudinea	The mean proportion of the total gut content volume accounted for by leeches.

### Data set 2.

#### Data set name

Bottom-up matrices_FW_v2.xlsx

#### Data format

Excel spreadsheet

#### Description

This dataset contains information on the food web topology (feeding links) and interaction strength (proportional contribution of diets to consumers) summarised in a predator (consumer)-prey (resources) matrix for 12 reservoirs in Singapore (Wilkinson et al. 2021). Each row in the matrix represents a resource/prey, while each column represents a consumer/predator. For each matrix element that is > 0, it expresses the relative contribution of each resource to the diet of its consumer.

**Data set 2.** 

Column label	Column description
benthic algae	Primary producer: refers to filamentous algae.
emergent macrophytes	Primary producer: for example, *Ludwigia ascendens*.
floating macrophytes	Primary producer: includes vegetation within floating wetlands, for example, cattail (genus: *Typha*) plants.
macrophytes	Primary producer: includes plants in the genera: *Hydrilla*, *Mayaca* and *Valisineria*.
periphyton	Primary producer: refers to encrusted algae/periphyton, scraped from rocks.
phytoplankton	Primary producer.
riparian grasses	Primary producer: includes all C4 plants that are typically grasses in the riparian zone.
riparian plants	Primary producer: includes all C3 plants in the riparian zone.
unknown producer	Primary producer: We were unable to collect phytoplankton (or other pelagic/planktonic producers from three reservoirs, so a node was created to simulate this. The values of this node were subsequently based on the zooplankton nodes in each of the three reservoirs (Grey et al. 2000, Post 2002, Matthews and Mazumder 2003).
Ampullariidae	Invertebrate family, predominantly Pomacea.
Bivalvia	Invertebrate class.
Conchostraca	Invertebrate suborder.
Decapoda	Invertebrate order.
Chironomidae	Invertebrate family.
Ephemeroptera	Invertebrate order.
Gastropoda	Invertebrate class.
Hemiptera	Invertebrate order.
Hirudinea	Invertebrate subclass.
Nassariidae	Invertebrate family, assassin snails.
Odonata	Invertebrate order.
Oligochaeta	Invertebrate subclass.
Ostracoda	Invertebrate class.
Trichoptera	Invertebrate order.
Copepoda	Invertebrate subclass.
AA	Fish species:* Aplocheilus armatus*.
AC	Fish species:* Amphilophus citrinellus*.
AH	Fish species:* Acarichthys heckelii*.
AS	Fish species:* Atractosteus spatula*.
BS	Fish species:* Barbonymus schwanefeldii*.
CA	Fish species:* Cyclocheilichthys apogon*.
CC	Fish species:* Cyprinus carpio*.
CG	Fish species:* Clarias gariepinus*.
Cichla	Fish genus:* Cichla *spp.
CL	Fish species:* Channa lucius*.
CM	Fish species:* Channa micropeltes*.
CO	Fish species:* Cichla orinocensis*.
COr	Fish species:* Chitala ornata*.
CS	Fish species:* Channa striata*.
CT	Fish species:* Cichla temensis*.
MU	Fish species:* Mayaheros urophthalmus*.
DC	Fish species:* Dermogenys collettei*.
DM	Fish species:* Datnioides microlepis*.
ES	Fish species:* Etroplus suratenss*.
GA	Fish species:* Geophagus altifrons*.
Gam	Fish species:* Gambusia affinis*.
GAu	Fish species:* Glossogobius aureus*.
Goby	Fish family: Gobiidae.
HB	Fish species:* Heterotilapia buttikoferi*.
HQ	Fish species:* Hyporhamphus quoyi*.
HR	Fish species:* Hemigrammus rodwayi*.
LR	Fish species:* Leptobarbus rubripinna*.
MC	Fish species:* Megalops cyprinoides*.
MJ	Fish species:* Monopterus javanensis*.
MZ	Fish species:* Macrognathus zebrinus*.
NN	Fish species:* Notopterus notopterus*.
OG	Fish species:* Osphronemus goramy*.
OH	Fish hybrid genus:* Oreochromis *spp. (hybrid).
OJ	Fish species:* Oryzias javanicus*.
OM	Fish species:* Oxyeleotris marmorata*.
OMo	Fish species:* Oreochromis mossambicus*.
ON	Fish species:* Oreochromis niloticus*.
OV	Fish species:* Osteochilus vittatus*.
PD	Fish species:* Pterygoplichthys disjunctivus*.
PMa	Fish species:* Parachromis managuense*.
PMot	Fish species:* Potamotrygon motoro*.
PP	Fish species:* Pterygoplichthys pardalis*.
PS	Fish species:* Parambassis siamensis*.
Pter	Fish genus:* Pterygoplichthys *spp.
RB	Fish species:* Rasbora boraptensis*.
RS	Fish species:* Rhinogobius similis*.
SF	Fish species:* Scleropages formosus*.
VM	Fish species:* Vieja melanura*.

### Data set 3.

#### Data set name

R_script.R

#### Data format

R script

#### Description

This file contains the R code to create the predation matrix and food web for a sample reservoir (Upper Peirce; Res 6). We used R version 3.5.2 to develop the code. Within R, the following packages were used and are necessary to create the predation matrices and food webs: simmr version 0.4.2 (Parnell 2020); NetIndices; ggplot2; and igraph.

**Data set 3.** 

Column label	Column description
R code	R code.

### Data set 4.

#### Data set name

MixingModelInputs_Res6.zip

#### Data format

Zip file of excel spreadsheets

#### Description

This folder contains both the source data (feeding links) and stable isotope ratios for both carbon and nitrogen isotopes for each taxa within the food web for a sample reservoir (Upper Peirce; Res 6). For each taxa, there will be two csv files, the first TaxaX.csv (listed by the taxa identifiers described above) with the SIA data for that taxa and the second TaxaX.Sources.csv with all the source data for that taxa.

**Data set 4.** 

Column label	Column description
Sheet 1: TaxaX.csv D13C	The d^13^C ratio for each individual of the specified taxa.
Sheet 1: TaxaX.csv D15N	The d^15^N ratio for each individual of the specified taxa.
Sheet 2: TaxaX.Sources.csv Sources	Taxa identifier of the prey source.
Sheet 2: TaxaX.Sources.csv Meand13C	Mean d^13^C ratio for the prey source.
Sheet 2: TaxaX.Sources.csv SDd13C	Standard deviation d^13^C ratio for the prey source.
Sheet 2: TaxaX.Sources.csv Meand15N	Mean d^15^N ratio for the prey source.
Sheet 2: TaxaX.Sources.csv SDd15N	Standard deviation d^15^N ratio for the prey source.
Sheet 2: TaxaX.Sources.csv tefd13C	^13^C trophic enrichment factor for the prey source.
Sheet 2: TaxaX.Sources.csv tefSDd13C	Standard deviation of^ 13^C trophic enrichment factor for the prey source.
Sheet 2: TaxaX.Sources.csv tefd15N	^15^N trophic enrichment factor for the prey source.
Sheet 2: TaxaX.Sources.csv tefSDd15N	Standard deviation of^ 15^N trophic enrichment factor for the prey source.
Sheet 2: TaxaX.Sources.csv conc13C	The weight of carbon recorded in the sample.
Sheet 2: TaxaX.Sources.csv conc15N	The weight of nitrogen recorded in the sample.

## Supplementary Material

1FA79DAE-1C25-57B2-80BA-BEBC1182F42A 10.3897/BDJ.10.e86192.suppl1 Supplementary material 1 R Script for creating a food web of reservoir 6 Data type R script for reproducing work presented in the paper. File: oo_577280.R https://binary.pensoft.net/file/577280 Clare L Wilkinson, Rayson B H Lim, Jia Huan Liew, Jeffrey J T Kwik, Claudia L Y Tan, Heok Hui Tan, Darren C J Yeo

4EA3E260-75C4-513C-8E7E-754518EBF572 10.3897/BDJ.10.e86192.suppl2 Supplementary material 2 Food web data for reservoir 6 Data type Food web File: oo_577284.zip https://binary.pensoft.net/file/577284 Clare L Wilkinson, Rayson B H Lim, Jia Huan Liew, Jeffrey J T Kwik, Claudia L Y Tan, Heok Hui Tan, Darren C J Yeo

## Figures and Tables

**Table 1.** Additional information on the 12 reservoirs in Singapore, including the number of transects sampled, sampling period, the type of reservoir (estuarine – reservoir has a tidal gate, but water is not saline; forest – predominantly surrounded by forest in the riparian zone; urban - predominantly developed riparian zone), mean pH and salinity and the year the reservoir construction was completed.

	**Latitude**	**Longitude**	**# of ** **transects**	**Sampling period**	**Type**	**Mean pH**	**Mean salinity ** **(ppt)**	**Year constructed**
Res 1	1°20.5'N	103°55.5'E	6	Sep–Oct 2015	Urban	8.24	0.101	1984
Res 2	1°17.2'N	103°52.0'E	6	Apr–May 2016	Estuarine	7.7	0.169	2008
Res 3	1°18.9'N	103°44.6'E	6	Jun–Jul 2015	Urban	8.27	0.141	1974
Res 4	1°24.2'N	103°53.2'E	6	Dec 2014–Feb 2015	Estuarine	-	-	2006
Res 5	1°23.4'N	103°55.0'E	6	Mar–Apr 2015	Estuarine	8.4	0.168	2011
Res 6	1°22.1'N	103°48.3'E	8	Dec 2015–Feb 2016	Forest	7.34	0.13	1974
Res 7	1°20.5'N	103°43.7'E	6	Jan–Mar 2017	Urban	7.4	0.141	1971
Res 8	1°22.2'N	103°49.4'E	6	Apr–May 2018	Forest	7.11	0.071	1910
Res 9	1°20.7'N	103°49.3'E	6	Jul–Aug 2017	Forest	6.56	0.031	1907
Res 10	1°24.2'N	103°48.0'E	8	Jan–Mar 2018	Forest	7.48	0.069	1969
Res 11	1°24.3'N	103°50.6'E	6	May–Jun 2018	Estuarine	8.19	0.093	1984
Res 12	1°25.5'N	103°44.4'E	8	Aug–Oct 2018	Estuarine	8.37	0.095	1975

**Table 2.** The records of all fish species and taxonomic groups included in the food webs across all 12 reservoirs, where "1" indicates that a taxon is present.

**Taxa**	**Res 1**	**Res 2**	**Res 3**	**Res 4**	**Res 5**	**Res 6**	**Res 7**	**Res 8**	**Res 9**	**Res 10**	**Res 11**	**Res 12**
**Basal sources**												
benthic algae	1		1	1	1		1		1	1	1	1
emergent macrophytes												1
floating macrophytes		1										
macrophytes								1	1	1	1	1
periphyton						1	1	1	1	1	1	1
phytoplankton	1	1	1	1	1	1		1		1	1	1
riparian grasses	1	1	1	1		1	1	1			1	1
riparian plants	1	1	1	1	1	1	1	1	1	1	1	1
unknown producer							1	1	1			
**Invertebrates**												
Ampullariidae	1		1	1			1			1	1	1
Bivalvia							1	1				1
Conchostraca								1	1	1		1
Decapoda	1	1	1	1	1	1	1	1	1	1	1	1
Chironomidae	1	1	1	1	1	1	1	1	1	1	1	1
Ephemeroptera	1							1		1	1	1
Gastropoda	1	1	1	1	1	1	1	1	1	1	1	1
Hemiptera								1	1	1	1	1
Hirudinea		1										
Nassariidae							1					1
Odonata	1		1	1	1	1	1	1	1	1	1	1
Oligochaeta							1					1
Ostracoda	1		1	1								1
Trichoptera						1			1			
Copepoda	1	1	1	1	1	1	1	1	1	1	1	1
**Fish**												
*Acarichthys heckelii*		1						1	1	1	1	1
*Amphilophus citrinellus*		1	1		1		1					
*Aplocheilus armatus*									1			
*Atractosteus spatula*												
*Barbonymus schwanefeldii*		1					1					
*Channa lucius*										1		
*Channa micropeltes*				1		1	1	1	1	1	1	1
*Channa striata*		1	1	1	1	1	1	1	1	1	1	1
*Chitala ornata*				1		1			1		1	1
*Cichla orinocensis*	1	1	1	1	1			1	1			
*Cichla *spp.							1			1	1	1
*Cichla temensis*	1	1				1		1	1			
*Clarias gariepinus*	1		1									
*Cyclocheilichthys apogon*									1			
*Cyprinus carpio*												
*Datnioides microlepis*	1											
*Dermogenys collettei*							1					1
*Etroplus suratensis*	1	1		1	1		1				1	1
*Gambusia affinis*							1			1		
*Geophagus altifrons*	1	1			1	1	1	1	1	1	1	1
*Glossogobius aureus*	1	1		1	1	1		1	1		1	1
Gobiidae										1		1
*Hemigrammus rodwayi*												1
*Heterotilapia buttikoferi*	1	1	1		1	1	1			1		
*Hyporhamphus quoyi*				1								
*Leptobarbus rubripinna*							1					
*Macrognathus zebrinus*											1	
*Mayaheros urophthalmus*		1		1			1					
*Megalops cyprinoides*	1											
*Monopterus javanensis*			1	1		1		1	1			
*Notopterus notopterus*			1							1	1	1
*Oreochromis mossambicus*		1			1							
*Oreochromis niloticus*		1		1	1		1					1
*Oreochromis *spp.(hybrid)	1				1							
*Oryzias javanicus*					1							
*Osphronemus goramy*					1	1	1	1		1	1	1
*Osteochilus vittatus*									1			
*Oxyeleotris marmorata*	1	1	1	1	1	1	1	1	1	1	1	1
*Parachromis managuense*			1									
*Parambassis siamensis*			1				1		1			1
*Potamotrygon motoro*								1		1	1	
*Pterygoplichthys disjunctivus*		1			1							
*Pterygoplichthys pardalis*		1										1
*Pterygoplichthys *spp.							1					
*Rasbora boraptensis*											1	
*Rhinogobius similis*	1		1	1	1							
*Scleropages formosus*						1		1	1	1		
*Vieja melanura*		1	1		1		1					
