# Supplementary figures and images for: Empirical food webs of 12 tropical reservoirs in Singapore
Source: Biodivers Data J. 2022 Sep 14;10:e86192. doi: 10.3897/BDJ.10.e86192 (PMC9848559; doi:10.3897/BDJ.10.e86192)

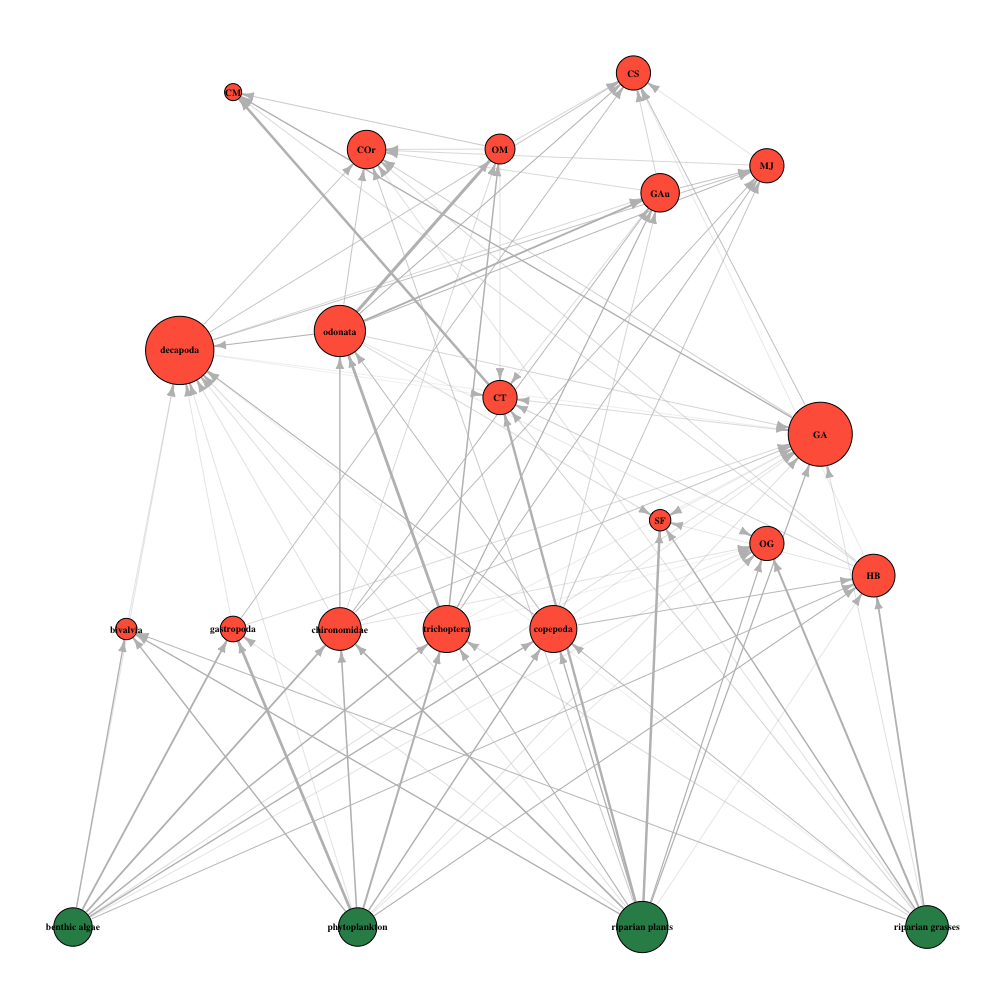

Supplement: Supplementary material 2 — Food web data for reservoir 6 [file bdj-10-e86192-s002.zip › MixingModelInputs_Res6/Reservoir 6 food web.tiff]
